# Exploring the Genetic Potential for Multi-Resistance to Rust and Other Coffee Phytopathogens in Breeding Programs

**DOI:** 10.3390/plants14030391

**Published:** 2025-01-28

**Authors:** Bruna Lopes Mariz, Eveline Teixeira Caixeta, Marcos Deon Vilela de Resende, Antônio Carlos Baião de Oliveira, Dênia Pires de Almeida, Danúbia Rodrigues Alves

**Affiliations:** 1 Instituto de Biotecnologia Aplicada à Agropecuária (BIOAGRO), Universidade Federal de Viçosa, Avenida Peter Henry Rolfs, s/n, Viçosa 36570-900, MG, Brazil; bruna.mariz@ufv.br (B.L.M.); denia.almeida@ufv.br (D.P.d.A.); danubia.alves@ufv.br (D.R.A.); 2 Embrapa Café, Parque Estação Biológica, Av. W3 Norte, Brasília 70770-901, DF, Brazil; marcos.resende@embrapa.br (M.D.V.d.R.); antonio.baiao@embrapa.br (A.C.B.d.O.)

**Keywords:** *Hemileia vastatrix*, coffee breeding, genetic resistance, molecular marker, gene pyramiding, morphoagronomic modeling

## Abstract

The application of marker-assisted selection in coffee breeding programs accelerates the identification and concentration of target alleles, being essential for developing cultivars resistant to multiple diseases. In this study, a population was developed from artificial crossings between Timor Hybrid and Tupi Amarelo, with the aim of promoting the pyramiding of resistance genes to the main diseases and pests of *Coffea arabica*: coffee leaf rust (CLR), coffee berry disease (CBD), cercospora, and leaf miner. Resistance was confirmed by nine molecular markers at loci associated with CLR (genes *SH3*, *CC-NBS-LRR, RLK*, *QTL-GL2*, and *GL5*) and with CBD (gene *Ck-1*). The resistance to CLR, cercospora, and leaf miner was evaluated using phenotypic diagrammatic scales. Mixed models estimated population superiority in 16 morphoagronomic traits over four agricultural years. The introgression of resistance alleles to CLR and CBD was identified in 98.6% of the population, with 29% showing pyramiding of five resistance genes. These pyramiding genotypes showed 100% resistance to the leaf miner and 90% to cercospora. The traits were grouped into univariate, bivariate, and trivariate repeatability models, with 11 significant ones. These results are indicative of genetic variability to be explored in the development of cultivars with multiple resistances and high agronomic potential.

## 1. Introduction

The development and use of resistant cultivars have proven to be the most suitable methods for sanitary control in crops, due to the cost–benefit ratio, efficacy, easy adoption by producers, as well as the low environmental impact. In coffee improvement programs, interspecific and intraspecific crossings have been carried out to introgress resistance genes into cultivars with agronomic characteristics of commercial interest [1,2,3,4]. Therefore, the importance of gene stacking is to obtain cultivars with durable multiple resistance to different pathogens as well as optimal beverage quality, high productivity, and morphoagronomic characteristics that facilitate phytotechnical management [5,6,7].

Among the most aggressive and pandemic diseases is coffee leaf rust (CRL), the causal agent is the fungus *Hemileia vastatrix* Berk. et Br., the coffee berry disease (CBD) caused by *Colletotrichum kahawae,* and cercosporiosis (CER) caused by *Cercospora coffeicola.* The most prevalent pest in the crops is the coffee leaf miner (CLM) caused by *Leucoptera coffeella*. These pathogenic agents specialized in coffee have competitive advantages due to being hemibiotrophic with easy dispersion, capable of attacking at any phenological stage and exhibiting a high adaptability to different microclimates [8,9,10,11,12,13,14].

The CLR can cause productivity losses of over 50% due to the premature dropping of leaves and drying out of productive branches, which creates energy deficits for the development of flower buds [15]. More than 120 cultivars of arabica coffee are registered, most of which have had their resistance surpassed by the fungus *H. vastatrix* [16]. This scenario reinforces the importance of ongoing research in identifying new sources of resistance and in pyramiding resistance genes [17,18,19].

Currently, it is known that at least nine dominant resistance genes to CLR are present in coffee plants of different species, which can act together or individually. The *S_H_1* to *S_H_5* genes have been identified in *C. arabica*, but they have already been replaced by CLR in several coffee cultivation areas. The *S_H_6* to *S_H_9* genes were detected in *C. canephora,* and the *S_H_3* gene was identified in *C. liberica* [5,20,21].

Some sources of resistance to CLR are known and used in coffee breeding programs; they are derived from Timor Hybrid (HdT), Icatu, BA series, and other Indian selections. The HdT is the only natural cross between *C. arabica* and *C. canephora*, and it possesses the *S_H_5* gene, derived from arabica, and the *S_H_6*, *S_H_7*, *S_H_8*, and *S_H_9* genes, derived from canephora [6,11,22,23]. Studies suggest the existence of two additional main resistance genes that have not yet been characterized, along with several others of lesser effect, which may or may not be associated with the genes *S_H_1*–*S_H_9*. These genes theoretically confer resistance to more than 50 races of *H. vastatrix* [17,24,25].

CBD has devastated many coffee plantations, especially on the African continent [26,27]. Productivity losses can reach 80% if no control measures are applied [28] and 100% in areas with heavy rainfall and high altitude [29]. So far, there are no reports of the disease in Latin America and Asia. However, CBD poses an imminent risk to coffee cultivation worldwide. There are various governmental efforts in preventive management to prevent its establishment in producing countries, as well as the development of resistant cultivars through preventive genetic improvement [10,26,30,31].

The CBD resistance in *C. arabica* is governed by three genes [32]. These genes are the *R* gene in the variety Rume Sudan, *T* gene in HdT, and a recessive *k*-gene found in both K7 and Rume Sudan. The *T* and *R* genes are dominant while the *k*-gene is recessive and only confers partial resistance to CBD in a homozygous state. The *R* locus has been reported to have multiple alleles (R1R1) in *C. arabica* variety Rume Sudan [31,32,33].

In addition to the main coffee diseases, CLR and CBD, plantations also face productive losses of up to 30% due to CER [13] and 50% due to CLR [9]. In advanced infections, there is leaf and lateral branch drop, accelerated maturation, and an increase in the incidence of defective grain formation [34,35]. So far, no resistance gene has been identified for CER and CLR, with resistance observed only through morphological markers and visual field analyses.

Molecular marker-assisted selection has been used in the genetic improvement of coffee plants to identify genes associated with resistance to CLR and CBD [36]. This approach is fundamental for characterizing resistance to the pathogen, even in the absence of its occurrence in the cultivation areas. Furthermore, it enables the understanding of inheritance dynamics and the genetic variability of populations intended for improvement [19,37].

In addition to molecular analysis, statistical methodologies applied to the agronomic traits of plants have been used to enhance the efficiency of selection in genetic improvement [38,39]. Mixed models allow for high accuracy in estimating variance components and genetic parameters, predicting gains from selection, and studying repeatability in perennial coffee plants [40]. These models enable the comparison of individuals over time and space, embedded in a complex data structure of morphoagronomic traits [41,42].

Despite advances in coffee breeding programs, the development of cultivars with multiple disease resistances is still a major challenge, especially due to the reliance on visual selection, which predominantly considers the phenotype of the plants [7,43]. This process is slow and often limited by the low efficiency in incorporating multiple genes for lasting resistance. Furthermore, there is a persistent lack of studies addressing the pyramiding of specific genes for simultaneous resistance to CLR, CBD, CER, and CLM in coffee cultivars, considering the complexity of the co-evolution of the pathosystem.

In this context, the present research is justified by the need to accelerate and improve genetic enhancement programs through the application of marker-assisted selection and robust data analysis using mixed models. These integrated approaches allow for the efficient identification and concentration of target alleles, optimizing time and resources for the development of superior cultivars. This study aims to develop and evaluate a population obtained through artificial hybridizations between cultivars of arabica coffee with sources of genetic resistance to CLR, CBD, CER, and the CLM. The main focus is the pyramiding of resistance genes and the identification of agronomically promising genotypes, contributing to overcoming current limitations and innovation in coffee genetic breeding.

## 2. Results

### 2.1. Assisted Selection by Molecular Markers for CLR and CBD

**Figure 1:**
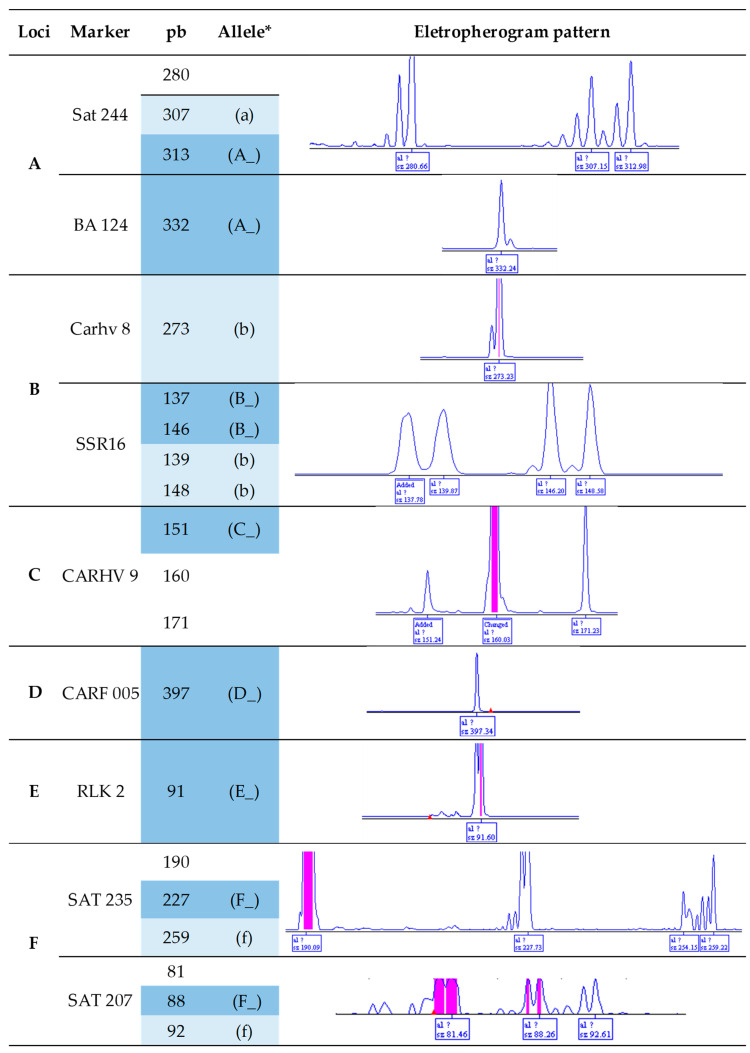
Description of the molecular markers for *Hemileia vastatrix* and *Colletotrichum kahawae*, covering the locus, the length of the generated electropherogram, and the identified alleles. * Electropherograms relevant for analysis are highlighted in blue, with dark blue representing dominant alleles and light blue representing recessive ones.

Molecular markers for CLR and CBD were developed for the analysis of results in agarose and polyacrylamide gels. These markers were integrated with fluorescent probes compatible with Sanger genotyping. All electropherograms detected in the marker region are presented in Figure 1, including the nonspecific ones.

The parents HdT MG 0357 and Tupi Amarelo IAC 5162 presented, respectively, the genotypes aaBBC-D-eeFF and aaBbccddE-Ff (Table 1). For the markers associated with locus B, 57.04% of the F_2_ individuals showed the dominant homozygous resistance allele, 33.80% were heterozygotes, and only 9.15% were recessive homozygotes (without the resistance allele). At locus C, the presence of the resistance allele (C_) was identified in 59.15% of the segregating progeny. In 74.65% genotypes of the population F2 was observed the presence of locus D. In 71.13% genotypes was observed the presence of locus E. No coffee plant in the F_2_ population presented the *S_H_3* gene.

Based on the amplification by SAT 235 and SAT 207 markers (locus F), it was observed that 56.34% individuals of the analyzed coffee plants have the *Ck-1* gene in homozygosity, 35.21% individuals in heterozygosity, and only 8.45% plants do not have the resistance gene for *C. kahawae*.

Only two individuals were homozygous recessive for all analyzed loci, representing 1.4% of the F_2_ population. This susceptibility can only be derived from the F_1_ hybrid C12P-8-B20-E5 (aaBbC_D_E_Ff), which is likely heterozygous for all loci. It is observed that in the genotype of F_1_ hybrid C12P-22-B20-E5 (aaBBC_D_eeFF), there is no possibility of double recessive mendelian segregation at loci B and F.

In the joint analysis for the four rust resistance loci (B, C, D, and E), 56 individuals had at least one resistance allele at each locus (B-C-D-E-), which represents 39.44% of the F_2_ population. Loci B and F were identified 45.07% as homozygous dominant for both loci (BBFF). Considering all loci/genes, 29% genotypes have dominant and double dominant alleles, with genotype BBC_D_E_FF.

The segregation test was significant only for loci D and E, with probabilities of 92% and 28%, respectively. For the other loci (A, B, C and F), the results were null, which may be attributed to the limited sample size, as the segregation test based on the chi-squared statistic is sensitive to the number of samples evaluated (Table 2).

### 2.2. Morphoagronomic Analyses

The variables that met the cut-off points and were useful in the analysis were 10 in 2018, 9 in 2020, 6 in 2021, and 11 in 2022 (Table 3). All evaluated traits showed significant genotypic variation in at least one year, indicating genetic variability in the population, except CF, which was null because it does not vary over the years. Based on h^2^a, 36% were in the range of 0.15 to 0.50, which are classified as moderate but are considered high in the scientific community for the evaluated quantitative traits. The estimated selective accuracy was higher than 0.60 in 53% of the traits and higher than 0.80 in 20% of the traits throughout all years, reflecting an overall average of 0.56.

Considering Y, in year 2018, it was significant with a low average (0.74 L/plant); in 2020, its h^2^a was null, with low accuracy, not significant, but with production four times higher than in 2018 (3.10 L/plant); in 2021, it was significant, with a low average and a marked discrepancy in performance between the best and worst genotypes; and in 2022, it was not significant and had a low average. The average VIG during the four evaluated years remained higher than six, which corresponds to coffee plants with adequate leafiness and homogeneous distribution of plagiotropic branches along the orthotropic branch. There was an increase in PH of coffee plants of about 39%, 6%, and 11% throughout the 2020, 2021, and 2022 harvests, respectively.

In the four evaluated years, the plants behaved resistant to coffee rust infection, with average scores on the diagrammatic scale below two. The CLR had high heritability in 2018 (0.39) and 2022 (0.50) and close to nullity in 2020 and 2021. In 2022, it was the year with the lowest incidences of CLR, CER, and CLM, with the highest h2a and Ac reported for these traits.

In the repeatability analyses (*r*), the cut-off point h^2^a > 0.03 was maintained, and non-significant character–year combinations were eliminated. Therefore, CF and SD characters could not be analyzed because they were not significant in any evaluated year. It is observed that the years 2018, 2020, 2021, and 2022 contributed with viable data for 10, 8, 4, and 7 traits, respectively (Table 4). Thus, it was possible to adopt triple repeatability models (3 years), double repeatability models (2 years), and univariate models (1 year).

The coefficients of r ranged from 0 to 0.59, with the majority of accuracies above 80%, and 11 significant traits/models. The heritability values of CD, QPB, LPB, and NNR were similar in h^2^g (~0.11) and h^2^ad (~0.06). For CLR and CLM, the genetic parameters were identical in *r* (0.15), h^2^g (0.14), and h^2^ad (0.08), and contradicting this pattern, CER had low and non-significant parameters by LRT.

### 2.3. Selection of Genotypes with Five-Gene Pyramiding for Resistance to CLR and CBD

In the F_2_ population, 29% of the genotypes exhibited pyramiding of five resistance genes, with loci B and F in homozygous dominant and loci C, D, and E containing at least one resistance allele (BBC_D_E_FF) for CLR and CBD (Table 5). The resistance alleles for *H. vastatrix* contributed to the phenotypic scores, using a diagrammatic scale, indicating resistance of all genotypes to coffee rust (average 1.58). These genotypes with double dominant genes for CLR and CBD were less affected by CER (score~2) and CLM (score~1.74), which may be an indication of cross-resistance. In general, all the genotypes with multiple resistance genes had high agronomic performance averages, considering the characteristics related to production (VIG = score 7, TF = score 3, PNR = 48, PCR = 0.64 metros, NNR = 20). The highest productive averages were from individuals 114 and 128, with 3.88 and 2.90 L per plant, respectively. These coffee plants had production up to three times higher when compared to the overall population average of 1.23 L per plant.

## 3. Discussion

In genetic breeding of coffee for disease resistance, genic pyramiding is the best way to obtain multiple loci that offer combined vertical resistance, thus limiting the infection of various pathogen races simultaneously [5,7]. The theory that “for every dominant resistance gene in the host, there is a dominant avirulence gene in the pathogen” was proposed by Flor in 1942 and is still accepted today to explain resistance in plants. Based on this theory, for a pathogen to overcome the resistance of genotypes, such as those identified in this work, that contain up to five resistance genes, it is necessary for mutations in five avirulence genes of the fungus to occur [44].

From the crossings between HdT MG0357 and Tupi Amarelo IAC 5162, genes for resistance to CLR and CBD were introduced (Table 1). According to the adoption of assisted selection, more than 60% of the genotypes of the segregating population had introgression of resistance to races I and II, identified at loci B and C. The combination of these loci (B and C) for these breeds allows for greater selection pressure exerted on the pathogen, making its infection with the genotype more difficult. In the Americas, 18 races of *H. vastatrix* have been reported, with race II being the most prevalent in the susceptible cultivars planted, which demonstrates the importance of the results obtained from this study [8,15]. The absence of the *S_H_3* resistance allele (locus A) in the F_1_ and F_2_ genotypes was predictable, as the population does not result from crosses with *C. liberica*, the source of this resistance [18]. Future backcrosses with this progeny may be carried out for the introgression of the *S_H_3* gene.

In 70% of the population, introgression of resistance was identified in loci D and E, corresponding to genes belonging to the *CC-NBS-LRR* and *LRR-RLK* families. These gene families correspond to the first line of defense of the plant against infection, as they are a diverse group of transmembrane receptors that can recognize molecular patterns associated with pathogens and activate an immune response [25,45]. The Coffea-*H. vastatrix* pathosystem is complex due to the constant co-evolution of races against the distinct defense mechanisms of plants [11,19,21]. Transcriptome and interactome studies of HdT identified target genes involved in a pre-haustorial defense response, associated with resistance to *H. vastatrix* [17].

Due to the crossings with the resistance source HdT, 90% of the F_2_ population exhibited resistance to CBD. CBD is a very aggressive disease with the potential to cause collapse in productive systems that have susceptible cultivars [27]. Research worldwide is conducted to monitor its migration and variations in virulence [29,30,46]. Even without reports of *C. kahawae* in South America [10,26], the introgression of the *Ck-1* gene into improved varieties is an important control measure in case the pathogen becomes established in the territory. This preventive improvement is only possible with the implementation of a molecular marker, to characterize and select resistance to CBD, without the presence of the pathogen [7,31].

Based on the genotypic results obtained, the individuals of the F_2_ population that exhibited pyramiding of five resistance genes (BBC_D_E_FF) were identified as the most promising for resistance to the diseases CLR and CBD (Table 5). These genotypes represent 29% of the population and exhibit a genetic combination with high potential for multiple resistance, making them priority candidates for advancement in subsequent generations.

With the exception of locus E, all other loci were genotyped using two molecular markers. The use of two or more markers at the same locus reinforces the reliability of the results, avoiding the selection of plants that have the marker but lost the resistance allele due to recombination [30,47,48]. Furthermore, studies that validated and applied these molecular markers confirmed their consistency, evidencing that their results are not impacted by sample size, as observed in the segregation test (Table 2) [24,25,30,47,49,50,51,52,53].

The REML/BLUP modeling used for the 16 morphoagronomic traits demonstrated effectiveness by providing statistical significance and high selective precision for the studies of genetic parameters and repeatability (Table 3 and Table 4). The LRT proved that most of the traits were significant with the combinations of crops, which justifies the repeatability models being the best to describe the behavior of time on perennial species. In general, the use of repeatability leads to higher accuracy, especially in situations of low heritability and repeatability simultaneously, as observed in Table 3 and Table 4.

For the traits FUC (0.19) and FMC (0.13), repeatability was high, considering that they are governed by many genes and highly influenced by environmental conditions from fruit formation to harvest. The low magnitudes of *r* in some traits show the lack of regularity in the repetition of behavior in the following evaluation years, consequently causing difficulties in the process of selecting superior genotypes based on few years of evaluation.

The number of measurements required for high accuracies generally requires several years of evaluations, which burdens the costs and time of coffee genetic improvement. It is estimated that to obtain 90% of the maximum accuracy, 17 measurements with heritability of 0.20 are required, which according to the literature is common for the traits of yield, stem diameter, plant height, crown diameter, and rust resistance. For traits with high heritability, for example, 0.50, the recommended number of replications is four to achieve 90% accuracy [38].

Another contributing factor to the low genetic parameters is the degree of relatedness between the parents since both are cultivars derived from HdT accessions. Previous studies on genetic diversity confirm that HdT MG 0357 and Tupi Amarelo IAC 5162 belong to divergent genetic groups but with a considered moderate genetic distance [54]. The genus Coffea has low genetic diversity, attributed to domestication and the perennial nature of the crop, being even more limited in *C. arabica* due to autogamy and the multiple genetic bottlenecks that occurred during polyploidization [1,2,3,4]. It also reports low genetic parameters in coffee arabica breeding programs for resistance to CLR, using artificial crossings between HdT and arabicas, and emphasizes the importance of morphoagronomic characterization combined with MAS to achieve better selective gains.

Although it may seem contradictory for the traits related to productivity to have more expressive genotypic variances than the actual production, this scenario is common due to the need for a greater number of evaluations, as reported in this and other studies [50,51]. Even so, there is an intrinsic genetic correlation among the characteristics related to productivity, such as Y, VIG, PH, CD, SD, QPB, LPB, and NNR.

The low severity of CER (scores~2) may be related to resistance genes that have not yet been characterized in these genotypes and also the good fertility of plants, which is a decisive factor for the low incidence of this disease [34,35].

Tolerance to CLM is observed in cultivars derived from the Sarchimor group, such as Tupi IAC 1669-33, which is an ancestral parent that contributed to the formation of the studied population [9]. Studies found that despite the high percentage of leaves damaged by CLM, the cultivar Tupi IAC 1669-33 demonstrated the ability to retain its leaves for a longer time, showing a better response to the attack [55]. Cultivars of *C. arabica* from HdT and resistant to rust may compromise the performance of the coffee leaf miner by prolonging the duration of the pupal development stage and reducing the size of the adults. The hypothesis is that crossings with the HdT could have modified the profile of nutrients and secondary metabolites in the leaf tissues, unfavorably for the development of the CLM [56].

Using the phenotypic averages obtained via the diagrammatic scale, it is possible to infer that the population is resistant to CLR throughout the years evaluated. These averages (close to 2) are related to hypersensitivity reactions in the leaf, which occur as an immune response to parasitic infection through encapsulation of haustoria in the intercellular spaces by lignification of cell walls and hypertrophy of plant cells, and/or increased activity of oxidative enzymes [43]. Therefore, the *H. vastatrix* fungus can penetrate the tissues, but it usually stops developing after the first haustoria formed, which is called post-haustorial resistance. Plants without any symptoms (score 1) can exhibit pre-haustorial resistance, which prevents the development of hyphae and the formation of haustoria in the tissues [15,20,21].

Despite the expected segregation in F_2_, the population behaving resistant to CLR is a strong indication that there was introgression of genes for this, corroborating with the molecular data that showed the pyramiding of resistance genes for *H. vastatrix*. No correlations were identified between the incidences of CLR, CER, and CLM among themselves and with Y, a fact that corroborates with [53]. However, [57] obtained positive and high correlations between CLR and CLM with heritability close to 90% and also did not obtain significant correlations between Y, CLR, and CLM.

A selection of 29% of the population with five pyramids of resistance genes to CLR and CBD, combined with field resistance to CER (average score of 2) and CLM (average score of 1.7), characterizes these genotypes as carriers of multiple resistances (Table 5). The FMC in this selection presented genotypes with variations in precocity, ranging from early to late (grades 2–5). This diversity enables the staggering of planting plots, optimizing the harvest due to maturation in stages. In addition, these genotypes contained a plant architecture for field distribution typical for arabica coffees, with averages of 1.30 m for PH, 0.35 m for SD, and 1.43 m for CD. These genotypes show promise for the advancement of generation, with selection gains aimed at increasing productivity, improving beverage quality, and monitoring multiple resistances.

The process of this genetic improvement program has already been ongoing for 15 years due to the hybridizations between the parent strains HdT MG 0357 and Tupi Amarelo IAC 5162. Considering the crossings made with their ancestors, it is estimated that the program has more than 35 years of history. Through robust statistics, morphoagronomic characterizations, and the adoption of marker-assisted selection in the upcoming generations, it is expected to accelerate the selection process. Thus, within a period of 10 years, it will be feasible to launch competitive cultivars that are highly resistant to *H. vastatrix* and other pathogens.

## 4. Materials and Methods

### 4.1. Prospecting for the Improvement Program

Artificial crosses were made between access of the HdT MG 0357, belonging to the Germplasm Bank of the Agricultural Research Corporation of Minas Gerais (EPAMIG, Minas Gerais, Brazil) and the lineage called Tupi Amarelo IAC 5162, originating from the Breeding Program of the Intituto Agronomico de Campinas (IAC, Campinas, Brazil).

The HdT MG 0357 is derived from the access HdT UFV 441-04, which was introduced in Brazil from the Center for Research into Coffee Rusts (CIFC), located in Oeiras, Portugal. These hybrids have good beverage quality, tall statures, medium maturation cycles, and high resistance to diseases and pests.

The Tupi Amarelo IAC 5162 line is a yellow-fruited Sarchimor, selected by the IAC. This line was developed from the identification of a plant with yellow fruits in a crop of the Tupi IAC 1669-33 cultivar. This cultivar originated from the hybrid CIFC H361-4, which is derived from the cross between Villa Sarchi CIFC 971/10 and HdT CIFC 832/2. Therefore, the line Tupi Amarelo IAC 5162 is probably derived from a natural cross between a plant of Tupi IAC 1669-33 and a coffee plant of the Catuaí Amarelo cultivar (Caturra × Mundo Novo). The Tupi IAC 1669-33 has red fruits, a high percentage of grains classified in sieve 16 and above, drink quality similar to Bourbon Vemelho, and moderately early and uniform maturation. It also has resistance to *H. vastatrix*, low stature, large fruits, and high productive potential.

The crossing was carried out with the main objective of pyramiding resistance genes to *H. vastatrix,* aiming for longer-lasting resistance to CLR and incorporating resistance to other diseases such as CBD. Other purposes of the cross were plant height reduction and taking advantage of the superior beverage quality potential of the HdT MG0357 accession, which was previously verified in various sensory analysis tests.

Previous studies were conducted on the genetic potential and combining ability of the cross HdT MG0357 × Tupi Amarelo IAC 5162 and the two F_1_ plants generated (C12P-8-B20-E5 and C12P-22-B20-E5) [54]. Subsequently, the F_1_ generation plants were self-pollinated to generate an F_2_ population composed of 142 genotypes, cultivated in Viçosa, MG, Brazil (20°44′28.4″ S, 42°50′53.9″ W).

**Figure 2:**
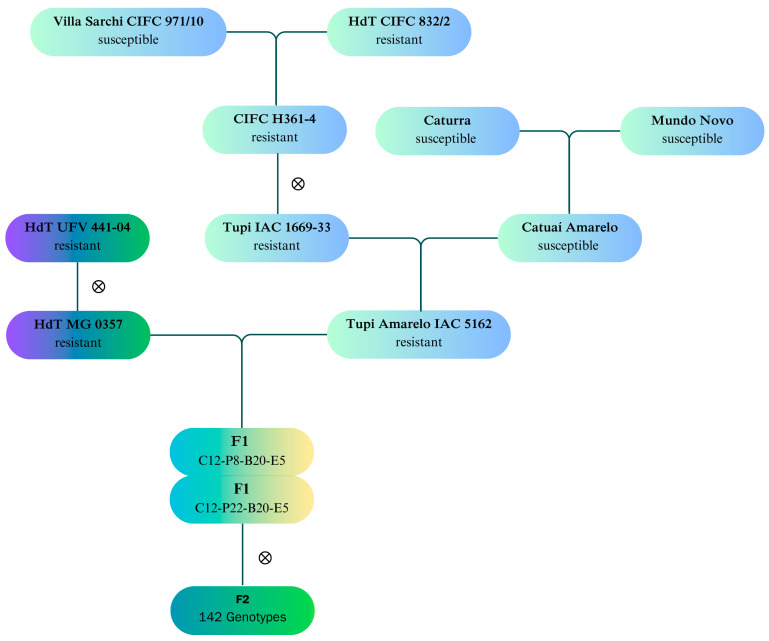
Prospecting of the coffee improvement program for durable multiple resistance to diseases and pests, resulting from the integration of genotypes for resistance to *Hemileia vastatrix*.

The design was in augmented blocks with a 3.0 × 0.80 m spacing. The cultivars Paraíso MG H419-1 and Catuaí Vermelho IAC 144 were used as controls, with three plants of each control per block. The control plant Paraíso MGH419-1 (Catuaí Amarelo IAC 30 × HdT UFV 445-46) was used for its high resistance to rust, short stature, medium maturation, high productivity, and cup quality. The Catuaí Vermelho IAC 144 (Caturra Amarelo IAC 476-11 × Mundo Novo IAC 374-19) was chosen for its high cultivation in Brazil (Figure 2).

### 4.2. Molecular Marker-Assisted Selection for CLR and CBD

The genetic material was extracted from the population according to the methodology described in [58]. The DNA concentration was quantified using Nanodrop (NanoDrop Technologies, Wilmington, EUA), and the quality was verified by 1% agarose gel electrophoresis.

For molecular marker-assisted selection, specific loci markers were used, previously identified as associated with genes that confer resistance to CLR and CBD. In the analysis of data for resistance to *H. vastatrix*, locus A, which corresponds to the *S_H_3* gene, was considered. We identified two markers SAT 244 and BA 124-12K, segregating at 0 cM with the *S_H_3* gene in the genetic mapping study of an F_2_ population (Matari × S.288) [47]. The S.288 line carried the *S_H_3* gene for resistance to CLR introgressed from *C. liberica*. Loci B and C corresponded to gene/QTL regions for resistance to races I, II, and pathotype 001 of *H. vastatrix.* Molecular markers associated with two groups of the genetic linkage map were identified. Markers SSR 16 and CaRHv8 were associated with Gene/QTL-GL2 at an approximate distance of 3 cM, and marker CaRHv9 was associated with 2.3 cm from Gene/QTL-GL5. These identified loci/QTL came from HdT (accession HdT-UFV 443-03), one of the main sources of resistance to coffee rust [36,59]. The D locus corresponds to the *CC-NBS-LRR* gene that confers resistance to *H. vastatrix*. In silico analysis, based on information generated by the Brazilian Coffee Genome Project [52], identified DNA sequences potentially involved in coffee disease resistance, for which they developed and validated the CARF 005 marker in the accession (HdT CIFC 832/2). The region amplified by the CARF 005 marker was confirmed as belonging to the HdT 832/2 accession by sequencing a BAC clone [25]. Furthermore, the authors [25], based on the availability of differential coffee clones, stated that it could be one of the unidentified SH genes that have not yet been supplanted (at least in Brazil) in HDT. The E locus corresponded to the Leucine-rich repeat receptor-like protein kinases (LRR-RLKs) gene family. This gene was identified by [24] through the sequencing of a BAC clone from accession 832/2. From the nucleotide sequence of the gene, the authors [24] developed the marker *LRR-RLK2* and confirmed by evaluating differential coffee clones that it could also be one of the unidentified S_H_ genes. Regarding the F locus, it corresponded to the resistance gene to another important coffee disease, CBD. This gene was named *Ck-1*, originating from the resistance source HdT, and was characterized using a genetic mapping approach. In this work, two SSR markers were identified as associated with the *Ck-1* gene [30]. The marker SAT 207 was mapped at 17.2 cM from the gene, while SAT 235 segregated at 0 cM with the *Ck-1* gene. These two markers were validated for use in assisted selection by [49] (Table 6).

All genotyping was conducted by capillary electrophoresis on an ABI 3130xl Genetic Analyzer—*AppliedBiosystems*.

- Assisted selection for the *S_H_3* gene—Locus A

For assisted selection, the molecular markers SAT 244 and BA 124-12K were used. The SAT 244 is codominant, and the BA 124-12K-f is dominant; so, when analyzed together, they can identify heterozygotes, dominant homozygotes, and recessive homozygotes.

The accesses CIFC H147/1, CIFC H153/2, and S.288/23 were used as controls carrying the resistance gene, and the cultivars Caturra Vermelho CIFC 19/1 and Catuaí Amarelo IAC 64 (UFV 2148/57) were used as non-carrying controls of the resistance gene to CBD.

In the amplification reaction of the fragments, 2 µL of genomic DNA at a concentration of 25 ng·µL^−1^ (50 ng) was used, 2.5 µL of PCR reaction buffer 1×, 1 µL of MgCl^2^ (2 mM), 0.25 µL of dNTP (0.1 mM), 5 µL of forward primer (0.4 μM), 5 µL of reverse primer (0.4 μM), 0.2 µL of Taq DNA polymerase enzyme (0.5 units), completing the volume to 25 µL with ultrapure water [47].

The amplification of the fragments consisted of denaturation at 95 °C for 5 min, 35 cycles of 94 °C for 45 s for denaturation, annealing at 52 °C for SAT 244 and 56 °C for BA 124-12K-f for 45 s, extension at 72 °C for 45 s, and final extension at 72 °C for 10 min.

- Assisted selection for QTL LG2—Locus B

For assisted selection, the molecular markers CaRHv8 and SSR 16 were used. The CaRHv8 is a dominant marker that identifies only the recessive allele, that is, the presence of allele amplification indicates being (b_) or being able to be homozygous recessive (bb) or heterozygous (Bb), and the absence of allele amplification indicates being homozygous dominant (BB). The SSR 16 marker presents a codominant pattern, which identifies homozygous and heterozygous individuals (BB, Bb, and bb).

As control, the HdT UFV 443-03 genitors were used as resistant and Catuaí Amarelo IAC 64 (UFV 2148/57) as susceptible, as they originated the F_2_ population of the genetic map where the loci/QTL associated with resistance to races I, II, and pathotype 001 were identified.

The reaction for CaRHv8 was performed with 2 µL of genomic DNA at a concentration of 25 ng·µL^-1^ (50 ng), 2 µL of PCR reaction buffer (1×), 0.8 µL of MgCl^2^ (2 mM), 0.3 µL of dNTP (0.15 mM), 1 µM of each primer, and 0.2 µL of Taq DNA polymerase enzyme (0.5 units), with a final volume of 20 µL. A program with denaturation at 95 °C for 5 min, 35 cycles of 94 °C for 30 s, annealing at 65 °C for 30 s, extension at 72 °C for 1 min, and final extension at 72 °C for 10 min was used.

The reaction for the SSR 16 marker was similar to CaRHv8; it only differs from the conditions of the CaRHv8 marker by using 0.4 µL of MgCl^2^ (0.6 mM). The cycling program had an initial denaturation phase at 94 °C for 2 min; 10 touchdown cycles at 94 °C for 30 s, with annealing temperature decreasing by 1 °C per cycle (66–57 °C) for 30 s, and extension at 72 °C for 30 s; followed by 30 cycles of denaturation at 94 °C, annealing at 57 °C, and extension at 72 °C, each step lasting 30 s. The final extension was performed at 72 °C for 10 min.

- Assisted selection for gene/QTL of *LG5*—Locus C

For assisted selection, the molecular marker CaRHv9 was used. It is a dominant marker that only identifies the dominant allele, that is, the presence of allele amplification indicates being (C_), which means it can be homozygous dominant (CC) or heterozygous (Cc), and the absence of allele amplification indicates being homozygous recessive (cc). We used the same controls, concentration of reagents, and amplification conditions as CaRHv8.

- Assisted selection for *CC-NBS-LRR*—Locus D

For assisted selection, the molecular marker CARF 005 was used. The CARF 005 is a dominant marker that allows the identification of genotypes D_ and dd. The controls used were HdT CIFC 832/2 and Caturra Vermelho CIFC 19/1, as resistant and susceptible, respectively.

The reaction conditions were 2 µL of genomic DNA at a concentration of 25 ng·µL^−1^ (50 ng), 2 µL of PCR reaction buffer (1×), 0.4 µL of MgCl^2^ (2 mM), 0.3 µL of dNTP (0.15 mM), 1 µM of each primer, 0.2 µL of Taq DNA polymerase enzyme (0.5 units), and water up to a final volume of 20 µL. The cycling program was 95 °C for 5 min for denaturation, 35 cycles of 94 °C for 30 s, 60 °C for 35 s for annealing, 72 °C for 1 min for extension, and finally strand closure at 72 °C for 10 min.

- Assisted selection for *HdT_LRR_RLK2*—Locus E

For assisted selection, the molecular marker LRR_RLK2 was used. The *LRR_RLK2* is a dominant marker capable of identifying genotypes E_ and ee. The controls used were the HdT CIFC 832/2 as resistant and Caturra Vermelho CIFC 19/1 as susceptible. The marker was amplified under the same reaction conditions and cycling program as the CARF 005 marker, except for the annealing temperature, which occurred at 66 °C for 30 s.

- Assisted selection for *Ck-1*—Locus F

For assisted selection, the molecular markers SAT 235 and SAT 207 were used. In the analysis of the population with these markers, the HdT UFV 377-15, UFV 440-10, and cultivar MGS Catiguá 3 were used as controls carrying the *Ck-1* gene. The susceptible controls used were Caturra Vermelho CIFC 19/1 and Catuaí Amarelo IAC 64 (UFV 2148-57).

In the amplification reaction of the fragments, 2 µL of genomic DNA at a concentration of 25 ng·µL^−1^ (50 ng), 2.5 µL of PCR reaction buffer 1×, 1 µL of MgCl^2^ (2 mM), 0.25 µL of dNTP (0.1 mM), 5 µL of forward primer (0.4 μM), 5 µL of reverse primer (0.4 μM), and 0.2 µL of Taq DNA polymerase enzyme (0.5 units) were used, completing the final volume of 25 µL. The amplification conditions consisted of a denaturation phase at 95 °C for 5 min; 35 cycles at 94 °C for 45 s; annealing at 50 °C for 45 s; extension at 72 °C for 45 s; and the final extension at 72 °C for 10 min.

The segregation of the markers was determined using the chi-squared test.

### 4.3. Evaluating Morphoagronomic Traits

In the fruit ripening stage, 16 phenotypic traits related to production, disease/pest resistance, and beverage quality were measured in the crops from 2018 to 2022 (Table 7).

Statistical analyses were performed using the Restricted Maximum Likelihood (REML) methodology to estimate the variance components by maximum likelihood. These components provide the basis for the Best Linear Unbiased Prediction (BLUP), used for predicting genetic values.

In the individual analyses, the model used was *y = Xr + Za + Wp + e*, where *y* is the data vector, *r* is the vector of repeat effects (assumed to be fixed) added to the overall mean, *a* is the vector of individual additive genetic effects (assumed to be random), *p* is the vector of plot effects, and *e* is the vector of errors or residuals (random). The uppercase letters represent the incidence matrices for the referred effects.

In the repeatability analysis, the model used was *y = Xm + Zg + Wb + Tp + e*, where *y* is the data vector, *m* is the vector of the effects of the measurement–repetition combinations (assumed to be fixed) added to the overall mean, *g* is the vector of genotypic effects (assumed to be random), *b* is the vector of block effects (assumed to be random), *p* is the vector of permanent environmental effects (in the case of plots) (random), and *e* is the vector of errors or residuals (random).

To adjust the model to more rigorous criteria and determine the significance of the characteristic, the following parameters were considered: individual accuracy greater than 0.5, *p*-value less than 0.25, and additive heritability greater than 0.03 [40]. All analyses were performed using Selegen REML/BLUP software version 2020 [60].

## Figures and Tables

**Table 1:** Molecular marker-assisted selection associated with coffee rust resistance: *S_H_3* gene (locus A); locus/QTL for resistance to races I, II, and pathotype 001 (loci B and C); *CC-NBS-LRR* (locus D); *HdT_LRR_RLK2* (locus E); and resistance to CBD, gene *Ck-1* (locus F).

N°	Individual *	Genotype	N°	Individual	Genotype	N°	Individual	Genotype
1	HdT MG 0357	aaBBC-D-eeFF	50	T22 B20 P20	aaBBccD-E-Ff	99	T23 B21 P33	aaBBC-D-E-FF
2	Tupi IAC 5162	aaBbccddE-Ff	51	T22 B20 P21	aaBbccD-eeFf	100	T23 B21 P36	aaBBC-D-E-Ff
3	C12-P8-B20-E5	aaBbC_D_E_Ff	52	T22 B20 P25	aabbccD-E-Ff	101	T23 B21 P37	aaBBC-ddE-FF
4	C12-P22-B20-E6	aaBBC_D_eeFF	53	T22 B20 P26	aabbccD-eeFf	102	T23 B21 P39	aaBbC-ddE-FF
5	T22 B19 P1	aaBBC-D-E-ff	54	T22 B20 P27	aaBBccddE-Ff	103	T23 B21 P40	aaBBC-ddE-FF
6	T22 B19 P3	aaBBC-ddE-Ff	55	T22 B20 P29	aabbccddeeff	104	T23 B21 P41	aaBBC-ddE-FF
7	T22 B19 P4	aaBbccddeeFf	56	T22 B20 P30	aaBbccD-eeFF	105	T23 B21 P42	aaBBC-ddE-FF
8	T22 B19 P5	aaBbccddeeff	57	T22 B20 P31	aaBbccD-E-Ff	106	T23 B21 P44	aaBBC-ddE-FF
9	T22 B19 P6	aaBbC-D-eeFf	58	T22 B20 P32	aaBBccD-E-FF	107	T23 B21 P45	aaBbC-ddE-FF
10	T22 B19 P7	aaBbC-D-eeFf	59	T22 B20 P34	aabbccD-eeFf	108	T23 B21 P46	aaBBC-ddE-FF
11	T22 B19 P9	aaBbC-D-eeFf	60	T22 B20 P35	aaBBccddeeff	109	T23 B21 P47	aaBBC-D-E-FF
12	T22 B19 P10	aaBbC-D-eeFF	61	T22 B20 P36	aaBbccD-E-Ff	110	T23 B21 P48	aaBBC-D-E-FF
13	T22 B19 P11	aaBbC-D-E-FF	62	T22 B20 P37	aaBBccddeeFf	111	T23 B21 P50	aaBBC-ddE-FF
14	T22 B19 P12	aaBbC-D-eeFF	63	T22 B20 P38	aaBbccD-E-Ff	112	T23 B22 P1	aaBBC-D-E-Ff
15	T22 B19 P13	aaBBC-D-E-Ff	64	T22 B20 P40	aaBBccD-E-FF	113	T23 B22 P3	aaBBC-D-E-FF
16	T22 B19 P15	aaBbC-D-eeFF	65	T22 B20 P42	aaBbccD-eeFf	114	T23 B22 P4	aaBbC-D-E-FF
17	T22 B19 P16	aaBbC-D-E-Ff	66	T22 B20 P43	aaBbccD-E-Ff	115	T23 B22 P5	aaBBC-ddE-FF
18	T22 B19 P17	aaBbC-D-eeFf	67	T22 B20 P44	aabbccD-E-Ff	116	T23 B22 P6	aaBBC-D-E-FF
19	T22 B19 P19	aaBBC-D-eeFf	68	T22 B20 P46	aaBbccddeeff	117	T23 B22 P7	aaBBC-D-E-FF
20	T22 B19 P20	aabbccddeeFf	69	T22 B20 P48	aabbccD-E-Ff	118	T23 B22 P8	aaBBC-D-E-Ff
21	T22 B19 P21	aabbccD-E-Ff	70	T22 B20 P49	aaBbccddE-FF	119	T23 B22 P9	aaBBC-D-E-FF
22	T22 B19 P22	aaBbccD-eeFf	71	T22 B20 P50	aaBBccD-E-FF	120	T23 B22 P11	aaBbC-D-E-Ff
23	T22 B19 P26	aaBBccddeeFf	72	T23 B21 P1	aaBBccD-E-FF	121	T23 B22 P12	aaBbC-D-E-FF
24	T22 B19 P35	aaBbccD-E-Ff	73	T23 B21 P2	aaBbccD-E-FF	122	T23 B22 P14	aaBBC-ddE-FF
25	T22 B19 P36	aaBBccD-eeFf	74	T23 B21 P3	aaBBccD-E-FF	123	T23 B22 P15	aaBbC-ddE-FF
26	T22 B19 P39	aaBbccD-E-FF	75	T23 B21 P4	aaBBC-D-E-FF	124	T23 B22 P17	aaBBC-D-E-FF
27	T22 B19 P40	aaBbccD-E-FF	76	T23 B21 P5	aaBBC-D-E-FF	125	T23 B22 P18	aaBBC-D-E-FF
28	T22 B19 P41	aaBbccddeeFf	77	T23 B21 P6	aaBBC-ddE-FF	126	T23 B22 P19	aaBBC-D-E-FF
29	T22 B19 P42	aaBbccddeeFf	78	T23 B21 P7	aaBbC-D-E-Ff	127	T23 B22 P20	aaBBC-D-E-FF
30	T22 B19 P43	aaBbccD-E-Ff	79	T23 B21 P9	aaBBC-D-E-FF	128	T23 B22 P21	aaBBC-D-E-FF
31	T22 B19 P44	aaBBccD-eeFf	80	T23 B21 P10	aaBbC-D-E-FF	129	T23 B22 P23	aaBBC-D-E-FF
32	T22 B19 P46	aaBBccddeeFF	81	T23 B21 P13	aaBbC-D-E-FF	130	T23 B22 P25	aaBBC-D-E-FF
33	T22 B19 P47	aaBbccD-E-Ff	82	T23 B21 P15	aaBBC-D-E-FF	131	T23 B22 P28	aaBBC-ddeeFF
34	T22 B19 P48	aaBBccD-E-Ff	83	T23 B21 P16	aaBBC-D-E-FF	132	T23 B22 P30	aaBBC-D-E-FF
35	T22 B19 P49	aaBbccD-eeFf	84	T23 B21 P17	aaBBC-D-E-FF	133	T23 B22 P34	aaBBccD-E-FF
36	T22 B19 P50	aaBbccddeeff	85	T23 B21 P18	aaBBC-ddE-FF	134	T23 B22 P35	aabbC-D-E-Ff
37	T22 B20 P3	aabbccD-eeFf	86	T23 B21 P19	aaBBC-ddE-FF	135	T23 B22 P37	aaBBC-D-E-FF
38	T22 B20 P4	aaBbccddeeff	87	T23 B21 P20	aaBBC-ddE-Ff	136	T23 B22 P38	aaBBC-D-E-FF
39	T22 B20 P5	aaBbccD-eeFf	88	T23 B21 P21	aaBBC-D_E-FF	137	T23 B22 P39	aaBBC-D-E-FF
40	T22 B20 P6	aaBBccD-eeFF	89	T23 B21 P22	aaBBC-D-E-FF	138	T23 B22 P40	aaBBC-D-E-FF
41	T22 B20 P7	aaBbccD-eeff	90	T23 B21 P24	aaBBC-D-E-FF	139	T23 B22 P41	aaBBC-D-E-FF
42	T22 B20 P8	aaBbccD-eeFf	91	T23 B21 P25	aaBBC-ddE-FF	140	T23 B22 P42	aaBbC-D-E-FF
43	T22 B20 P10	aaBbccddeeff	92	T23 B21 P26	aaBBC-D-E-FF	141	T23 B22 P43	aaBBC-D-E-FF
44	T22 B20 P11	aaBbccD-eeFf	93	T23 B21 P27	aaBBC-D-E-FF	142	T23 B22 P44	aaBBC-D-E-FF
45	T22 B20 P12	aabbccddeeff	94	T23 B21 P28	aaBBC-D-E-FF	143	T23 B22 P45	aaBBC-D-E-FF
46	T22 B20 P13	aabbccD-E-Ff	95	T23 B21 P29	aaBBC-D-E-FF	144	T23 B22 P46	aaBBC-D-E-FF
47	T22 B20 P15	aaBBccD-E-Ff	96	T23 B21 P30	aaBBC-D-E-FF	145	T23 B22 P49	aaBBC-D-E-FF
48	T22 B20 P17	aabbccD-eeff	97	T23 B21 P31	aaBBC-D-E-FF	146	T23 B22 P50	aaBbC-D-E-ff
49	T22 B20 P18	aaBBccD-eeFf	98	T23 B21 P32	aaBBC-D-E-FF	147	Paraíso H419-1	aaBBccddeeff
						148	Catuaí Vermelho	aabbccddeeFf

* Treatment 22, Block 19, Plant 1.

**Table 2:** Chi-squared segregation test for loci related to resistance to coffee rust: *S_H_3* gene (locus A), locus/QTL for resistance to races I, II, and pathotype 001 (loci B and C); *CC-NBS-LRR* (locus D); *HdT_LRR_RLK2* (locus E); and resistance to CBD, gene *Ck-1* (locus F).

Genetic Loci	Expected Segregation	Degrees of Freedom	Chi-Squared	Probability
A	1:2:1	2	426	0
B	1:2:1	2	80.03	0
C	3:1	1	19.01	0
D	3:1	1	0.01	92.28
E	3:1	1	1.14	28.65
F	1:2:1	2	77.55	0

**Table 3:** Estimated genetic parameters for the evaluated morphoagronomic traits.

Year	2018			2020			2021			2022		
Genetic Parameter	*h^2^a*	*Ac*	*μ*	*h^2^a*	*Ac*	*μ*	*h^2^a*	*Ac*	*μ*	*h^2^a*	*Ac*	*μ*
**Y**	0.25 *	0.80	0.74	-	0.14	3.10	0.04 *	0.56	0.96	-	0.14	0.52
**VIG**	0.09 *	0.76	6.33	0.04 *	0.67	6.66	-	0.27	6.40	0.03 *	0.55	6.63
**PH**	0.05 *	0.69	85.32	0.04 *	0.68	139.68	0.07 *	0.73	148.05	-	0.56	166.92
**FS**	0.05 *	0.67	2.97	0.08 *	0.74	3.08	-	0.10	2.81	-	0.11	2.82
**SD**	-	0.13	3.01	-	0.61	5.12	-	0.56	60.46	0.04 *	0.66	72.51
**CD**	0.18 *	0.81	106.15	0.16 *	0.80	138.12	-	0.44	144.73	0.04 *	0.65	153.01
**QPB**	-	0.23	34.89	-	0.16	52.94	-	0.52	41.83	0.11 *	0.76	60.91
**LPB**	0.12 *	0.78	46.33	0.25 *	0.83	63.16	-	0.15	71.80	-	0.55	62.37
**NNR**	-	0.10	12.90	-	0.41	23.69	-	0.17	24.85	0.11 *	0.77	19.31
**CLR**	0.39 *	0.83	1.61	0.07 *	0.67	1.98	-	0.26	1.91	0.50 *	0.84	1.58
**CER**	-	0.12	1.84	0.09 *	0.75	2.41	0.09 *	0.76	2.15	0.13 *	0.73	1.80
**CLM**	0.07 *	0.71	1.58	-	0.27	2.40	0.19 *	0.80	1.66	0.39 *	0.83	1.40
**CS**	0.44 *	0.85	1.90	0.44 *	0.85	1.90	0.44 *	0.85	1.90	0.44 *	0.85	1902.00
**CF**	-	0.48	1.44	-	0.48	1.44	-	0.48	1.44	-	0.48	1436.00
**FMC**	-	0.20	2.69	0.12 *	0.78	3.12	-	0.33	2.83	0.13 *	0.74	2.79
**FUC**	0.07 *	0.63	2.16	-	0.32	2.72	0.03 *	0.59	2.63	0.29 *	0.83	2.83

*h^2^a*: Individual additive heritability; *Ac*: accuracy; *µ*: average; *: significant 5%; **Y**: yield; **VIG**: vegetative vigor; **PH**: plant height; **FS**: fruit size; **SD**: stem diameter; **CD**: canopy diameter; **QPB**: quantity of productive branch; **LPB**: length of productive branch; **NNR**: number of nodes in the reproductive branch; **CLR**: coffee leaf rust severity; **CER**: cercosporiosis severity; **CLM**: coffee leaf miner infestation; **CS**: color of the sprout; **CF**: color of ripe fruit; **FMC**: fruit maturation cycle; and **FUC**: fruit uniformity cycle.

**Table 4:** Repeatability and its estimated genetic parameters for the morphoagronomic traits.

Genetic Parameter	Years	*r*	*h^2^g*	*Vg*	*Ve*	*h^2^ad*	*Ac-fam*	*Acc-Ind*	LRT
**Y**	2018	-	0.25	0.20	0.56	0.18	0.80	0.91	6.51 **
**VIG**	2018.2020	0.23	0.07	0.09	1.00	0.04	0.76	0.78	0 **
**PH**	2018.2021	0.01	0.00	10.84	1325.85	0.00	0.54	0.54	0.46 ^ns^
**FS**	2018.2020	0.06	0.02	0.00	0.19	0.01	0.61	0.62	0.48 ^ns^
**CD**	2018.2020	0.13	0.11	83.74	673.11	0.06	0.81	0.85	3.72 *
**QPB**	2022	-	0.11	-	-	0.06	0.77	0.81	2.36 *
**LPB**	2018.2020	0.12	0.12	30.72	226.66	0.07	0.82	0.86	5.45 **
**NNR**	2022	-	0.11	-	-	0.06	0.77	0.81	2.36 *
**CLR**	2018.2020.2022	0.15	0.14	0.05	0.31	0.08	0.84	0.89	13.08 **
**CER**	2020.2021.2022	0.08	0.02	0.01	0.41	0.01	0.62	0.63	0.27 ^ns^
**CLM**	2018.2021.2022	0.15	0.14	0.04	0.27	0.08	0.83	0.88	11.68 **
**CS**	2018.2020.2021	0.59	0.37	0.07	0.07	0.45	0.84	1.07	13.93 **
**FMC**	2020.2022	0.13	0.09	0.07	0.63	0.05	0.78	0.81	3.65 *
**FUC**	2018.2022	0.19	0.19	0.12	0.51	0.12	0.83	0.90	13.17 **

*r*: Repeatability of individual installments; *h^2^g:* genotypic heritability; *Vg*: genotypic variance; *Ve*: residual variance; *h^2^ad*: additive heritability; *Ac-Fam*: accuracy by PEV; *Ac-Indiv*: individual accuracy; LRT: Likelihood Ratio Test; * significance at 1%, ** significance at 5%, and ^ns^ not significant; **Y**: yield; **VIG**: vegetative vigor; **PH**: plant height; **FS**: fruit size; **SD**: stem diameter; **CD**: canopy diameter; **QPB**: quantity of productive branch; **LPB**: length of productive branch; **NNR**: number of nodes in the reproductive branch; **CLR**: coffee leaf rust severity; **CER**: cercosporiosis severity; **CLM**: coffee leaf miner infestation; **CS**: color of the sprout; **CF**: color of ripe fruit; **FMC**: fruit maturation cycle; and **FUC**: fruit uniformity cycle.

**Table 5:** Genotypes with high agronomic performance and gene pyramiding for resistance to CLR and CBD (BBC_D_E_FF).

Nº	Y	VIG	PH	FS	SD	CD	QPB	LPB	NNR	CLR	CER	LM	CS	CF	FMC	FUC
73	2.50	8	141	3	44	164	56	66	19	2	2	2	2	1	3	3
74	1.80	8	117	3	4	60	48	60	17	1	2	2	2	1	3	3
77	0.85	7	130	3	39	140	47	60	15	2	2	2	2	1	3	2
80	1.68	8	135	3	36	159	51	66	22	2	2	2	2	3	3	3
81	1.78	7	138	3	35	153	56	73	24	2	2	2	2	1	3	3
82	1.05	7	128	3	35	134	56	62	20	2	2	2	2	1	3	3
86	1.43	7	149	3	35	144	57	56	21	2	2	2	2	2	3	3
87	2.65	8	133	3	48	177	50	64	19	2	2	2	2	1	3	3
88	1.78	7	122	3	40	139	50	64	21	2	2	2	2	3	3	3
90	2.33	7	132	3	39	142	48	65	22	2	2	2	2	1	3	3
91	1.43	6	130	3	34	136	53	56	18	2	2	2	2	2	3	3
92	2.05	8	145	3	47	183	55	82	24	2	2	2	2	1	4	3
93	1.50	7	136	3	36	163	48	69	21	2	2	2	2	1	3	3
94	0.78	6	123	2	33	134	43	58	21	2	3	2	2	1	3	3
95	2.88	8	141	3	41	164	55	73	25	2	2	2	2	2	3	3
96	1.30	7	135	3	35	149	46	69	21	2	2	2	3	1	3	3
97	2.33	7	129	3	36	147	54	65	23	2	2	2	2	1	3	2
107	0.08	7	134	2	46	147	49	66	20	2	2	2	2	1	3	3
108	1.48	7	121	3	31	144	52	66	25	2	2	2	2	1	3	3
111	1.58	7	92	3	28	133	27	57	20	2	2	2	2	1	4	3
114	3.88	8	172	3	39	188	61	77	24	2	2	2	2	1	4	3
115	1.75	8	146	3	35	164	64	77	24	2	2	2	2	1	3	2
117	0.75	7	133	3	37	151	54	60	19	2	2	2	2	2	3	3
122	1.90	7	146	3	37	147	44	63	22	1	2	2	2	1	3	3
123	0.10	5	103	3	26	116	26	48	11	2	3	1	2	1	3	3
124	0.10	8	156	3	40	169	55	70	21	2	3	2	2	2	4	3
125	1.28	7	108	3	28	131	39	61	19	2	2	2	2	1	3	2
126	1.05	6	117	3	45	119	43	46	14	2	3	1	2	1	3	3
127	0.30	6	107	3	41	106	38	50	18	2	2	2	2	1	2	2
128	2.90	7	115	3	31	138	43	72	22	2	2	2	2	1	3	3
130	1.15	7	120	3	39	131	45	53	18	2	2	2	2	1	3	2
133	0.07	6	128	3	32	147	48	66	22	2	2	1	2	1	4	4
134	1.20	7	143	3	30	157	53	66	24	2	2	2	2	1	3	3
135	0.23	8	156	3	42	172	60	78	23	1	2	2	2	1	5	4
136	1.20	7	133	3	34	149	46	66	19	2	2	2	2	1	3	3
137	1.00	6	137	3	44	154	54	71	21	2	2	2	2	2	3	3
109	0.75	7	129	3	30	125	36	65	20	2	2	2	2	1	4	3
140	0.10	6	78	3	3	100	30	40	11	1	2	1	2	1	3	2
141	0.80	7	146	3	36	141	45	68	23	2	2	2	2	1	3	2
142	0.90	6	139	3	33	143	43	72	22	1	2	2	2	1	4	3
143	0.58	6	101	3	26	122	30	62	19	2	2	2	2	2	3	3
μ	1.35	6.81	129.74	2.88	34.72	143.33	47.51	64.0	20.23	1.58	1.99	1.74	2.02	1.27	3.07	2.76

Y: Yield; VIG: vegetative vigor; PH: plant height; FS: fruit size; SD: stem diameter; CD: canopy diameter; QPB: quantity of productive branch; LPB: length of productive branch; NNR: number of nodes in the reproductive branch; CLR: coffee leaf rust severity; CER: cercosporiosis severity; CLM: coffee leaf miner infestation; CS: color of the sprout; CF: color of ripe fruit; FMC: fruit maturation cycle; FUC: fruit uniformity cycle; and µ: average.

**Table 6:** Description of the molecular markers are associated with genes that confer resistance to *Hemileia vastatrix* and *Colletotrichum kahawae*.

Resistance	Locus	Gene	Marker	Type	Distance (cM)	Tag	Primers	T (°C)	Reference
*Hemileia* *vastatrix*	**A**	*S_H_3*	SAT 244	SSR	0	Codominant	F:GCATGTGCTTTTTGATGTCGT R:GCATACTAAGGAATTATCTGACTGCT	52	[47,49]
			BA-124 -12K-f	SCAR	0	Dominant	F:TGATTTCGCTTGTTGTCGAG R: TGCAGATTGATGGCACGTTA	56	
	**B**	*Gene/QTL-GL2*	CaRHv8	SCAR	3	Dominant	F:CCTTCTAGTGTTACCGAGGA R: CTTAGCGCCATGAATAGCCA	65	[59]
			SSR 016	SSR	3.7	Codominant	R:CCACACAACTCTCCTCATTC F:ACCCGAAAGAAAGAACCAAG	65	[48]
	**C**	*Gene/QTL-GL5*	CaRHv9	SCAR	2.3	Dominant	F:TGATGAAGAAGAGCGCATAGC R:GTCTAAGACCAGAATCAGATGG	65	[59]
	**D**	*NB-ARC* e *LRR*	CARF 005	Functional	.	Dominant	F:GGACATCAACACCAACCTC R:ATCCCTACCATCCACTTCAAC	60	[25,52]
	**E**	*HdT_LRR_RLK2*	RLK2	Functional	.	Dominant	F:GCTCACAGGTCCGATTCCTCTG R:TTTGGGAATAGGCCCGGAAAGA	60	[24]
*Colletotrichum kahawae*	**F**	*Ck-1*	SAT 235	SSR	0	Codominant	F:TCGTTCTGTCATTAAATCGTCAA R: GCAAATCATGAAAATAGTTGGTG	50	[30,49]
			SAT 207	SSR	17.2	Codominant	F:GAAGCCGTTTCAAGCC R: CAATCTCTTTCCGATGCTCT	50	

QTL: Quantitative Trait Locus; SSR: simple sequence repeat; SCAR: sequence characterized amplified region; and CAPS: cleaved amplified polymorphic sequence.

**Table 7:** Methodology for evaluating the main morphoagronomic traits of coffee.

TRAITS		
	**Y**	**Yield**
		Estimated in liters per plant
	**VIG**	**Vegetative vigor**
		Evaluated on a scoring scale ranging from 1 (minimum vigor) to 10 (maximum vigor)
	**PH**	**Plant height**
		Measured in the main orthotropic branch, from the soil surface to the final point of branch growth
	**FS**	**Fruit size**
		1 = tiny, 2 = small, 3 = medium, 4 = big, and 5 = large
	**SD**	**Stem diameter**
		Measured with the aid of a digital caliper, in the region of the plant’s stem (+ or −5 cm from the surface of the soil)
	**CD**	**Canopy diameter**
		Measured in the transverse direction to the planting line, measuring the largest projection of the coffee tree canopy
	**QPB**	**Quantity of productive branch**
		Number on the main stem
	**LPB**	**Length of productive branch**
		Measurement in the middle third of a representative plagiotropic branch of the plant
	**NNR**	**Number of nodes in the reproductive branch**
		Number of nodes of the representative plagiotropic branch of the plant measured in LPB
	**CLR**	**Coffee leaf rust severity**
		1—Absence of pustules and hypersensitivity reactions
		2—Few leaves with pustules without spores and hypersensitivity reactions
		3—Few pustules with high spore production and poorly distributed
		4—Medium content of pustules per leaf, with high spore production and well distributed throughout the plant
		5—High quantity of pustules, spore production, and plant defoliation
		Note: Plants with a score of 1 or 2 = resistant and 3 to 5 = susceptible
	**CER**	**Cercosporiosis severity**
		1—Leaf without cercospora symptoms
		2—Low incidence of cercospora lesions on the leaves
		3—Medium incidence of small-diameter cercospora lesions on the leaves
		4—High incidence of large-diameter cercospora lesions on the leaves
		5—Severity of cercospora on leaves with presence of necrosis
		Note: Plants with a score of 1 or 2 = resistant and 3 to 5 = susceptible
	**CLM**	**Coffee leaf miner infestation**
		1—Immune leaves, without any injury
		2—Leaves with few sharply shaped lesions
		3—Leaves with few and small lesions
		4—Leaves with moderate infestation and typical lesions with live larvae
		5—Leaves with severe infestation and typical lesions with live larvae
	**CS**	**Color of the sprout**
		1—Green; 2—light bronze; 3—bronze; and 4—dark bronze
	**CF**	**Color of ripe fruit**
		1—Green; 2—yellow; and 3—orange
	**FMC**	**Fruit maturation cycle**
		1—Early; 2—medium to early; 3—medium; 4—medium to late; and 5—late
	**FUC**	**Fruit uniformity cycle**
		1—Uniform; 2—moderately uniform; 3—moderately non-uniform; and 4—non-uniform

## Data Availability

In this manuscript, the molecular markers used in the analysis are previously available in the literature and referenced in the manuscript. The datasets generated during and/or analyzed during the current study are available from the corresponding author on reasonable request.
